# Propentofylline Targets TROY, a Novel Microglial Signaling Pathway

**DOI:** 10.1371/journal.pone.0037955

**Published:** 2012-05-23

**Authors:** Valerie L. Jacobs, Yingna Liu, Joyce A. De Leo

**Affiliations:** 1 Department of Pharmacology and Toxicology, Dartmouth Medical School, Hanover, New Hampshire, United States of America; 2 Neuroscience Center at Dartmouth, Lebanon, New Hampshire, United States of America; 3 Emmanuel College, Boston, Massachusetts, United States of America; University of Michigan School of Medicine, United States of America

## Abstract

Glioblastoma multiforme (GBM) is the most common and aggressive primary brain cancer, with a median survival of less than 2 years after diagnosis with current available therapies. The tumor microenvironment serves a critical role in tumor invasion and progression, with microglia as a critical player. Our laboratory has previously demonstrated that propentofylline, an atypical methylxanthine with central nervous system glial modulating and anti-inflammatory actions, significantly decreases tumor growth in a GBM rodent model by preferentially targeting microglia. In the present study, we used the CNS-1 rat glioma model to elucidate the mechanisms of propentofylline. Here we demonstrate that propentofylline targets TROY, a novel signaling molecule up-regulated in infiltrating microglia, and not macrophages, in response to CNS-1 cells. We identify Pyk2, Rac1 and pJNK as the downstream signaling molecules of TROY through western blot analysis and siRNA transfection. We demonstrate that inhibition of TROY expression in microglia by siRNA transfection significantly inhibits microglial migration towards CNS-1 cells similar to 10 µM propentofylline treatment. These results identify TROY as a novel molecule expressed in microglia, involved in their migration and targeted by propentofylline. Furthermore, these results describe a signaling molecule that is differentially expressed between microglia and macrophages in the tumor microenvironment.

## Introduction

Glioblastoma mutiforme (World Heath Organization [WHO] grade IV astrocytoma) is the most common and aggressive primary brain cancer, and despite current therapies, median survival is less than 2 years [1], [2]. The tumor microenvironment plays a critical role in glioma tumor growth. Microglia are a resident monocyte population in the central nervous system (CNS), which play a critical role in tumor initiation, invasion and growth [3], [4], [5], and together with macrophages, make up as much as 30% of the glioma tumor mass [3]. Glioma tumor cells actively recruit microglia and peripheral macrophages to the tumor site [6], [7]. Once there, these cells can secrete cytokines such as matrix metalloproteinases (MMPs), which help increase glioma invasion [8], [9]. The underlying mechanisms involved in microglial response to glioma are not completely understood, however, several molecules have been identified.

One class of molecules involved in microglia and glioma cell adhesion, migration and invasion are integrins [10]. Integrins are a family of heterodimeric transmembrane proteins composed of two subunits which mediate cell adhesion to the extra cellular matrix (ECM) [11]. Tumor Necrosis Factor Receptors (TNFR) are another large family of molecules often over expressed in glioma and involved in microglial signaling [12], [13], [14]. One TNFR in particular, TNFRSF19/TROY (Tumor Necrosis Factor Receptor of mouse embryo), has been recently reported to be over expressed in glioma and regulates migration of glioma cells through integrin signaling pathways [15]. In humans, TROY is minimally expressed in the brain with little to no expression in peripheral organs. Functions attributed to TROY include forming a complex with NgR and LINGO-1 in the peripheral nervous system to inhibit neurite outgrowth in adult mice, mediation of osteoblast versus adipocyte differentiation of human multipotent mesenchymal stromal cells and promoting glioblastoma cell invasion [16], [17], [18]. However, little is known about TROY's possible role in immune cells.

Propentofylline (PPF) is an atypical synthetic methylxanthine [1-(50-oxohexyl)-3-methyl-7-propylxanthine]. It has been studied extensively in several CNS disease animal models of stroke, opioid tolerance, and acute and chronic pain [19]. An important clinical feature of PPF is its minimal adverse side-effect profile, demonstrated in multiple clinical trials [19]. Known mechanisms include inhibition of cyclic AMP (cAMP) and cyclic GMP phosphodiesterases and action as a weak antagonist of the adenosine A1 receptor [20], [21]. More generally, PPF is a glial modulator with direct actions on microglia and astrocytes. We have previously demonstrated that PPF treatment significantly decreases tumor growth and increases survival in the CNS-1 rodent glioma model [9]. PPF did not cause apoptosis or decrease proliferation of CNS-1 tumor cells, but directly targeted microglia. Using *in vitro* methods we demonstrated that PPF decreased microglial migration towards CNS-1 tumor cells and decreased MMP-9 expression. A decrease in microglia MMP-9 expression was also determined in human glioma tissue samples. The effects of PPF were shown to be specific to microglia and not peripheral macrophages.

With the failure of single marker targeted therapies, a GBM therapy that targets glioma's invasive potential as well as the tumor promoting microenvironment are promising options. In this report, we sought to further study and identify the mechanisms of action of PPF. Here we demonstrate that PPF treatment decreases TROY expression and ultimately decreases downstream signaling molecules Pyk2, Rac1 and pJNK expression in microglia. Herein, we present TROY as a novel molecule increased in microglia in response to CNS-1 tumor cells, which further drives microglial migration. Finally, we demonstrate differential expression of TROY in CNS resident microglia versus infiltrative monocytes/macrophages. These results are profound, since microglia and peripheral macrophages share several of the same surface markers, and as such, these cells are rarely distinguished when studying the role of immune cells in the GBM tumor environment. In summary, TROY is a novel molecule involved in microglial migration, which is targeted by PPF.

## Materials and Methods

### Animals and Cell Lines

This work was approved by the Dartmouth College Institutional Animal Care and Use Committee (IACUC protocol 05-07-09). All efforts were made to minimize the number of animals used and their suffering. We used adult male Lewis Rats (250–300 g) for all animal studies (Harlan Laboratories, Indianapolis, IN). For tumor cell studies, we used the CNS-1 cell line - a rat glioma cell line, (generously donated by Dr. William F. Hickey, Dartmouth Medical School, Hanover, NH) [22]. IACUC protocols were not established during the time CNS-1 cells line was developed for citation purposes (1990), however, the highest ethical standards in animal care were upheld during use of these animals. The cell line has been tested within the last year by verification of GFAP expression with immunohistochemistry. Propentofylline was purchased from Toronto Research Chemicals (North York, ON).

### Cell Culture

Highly purified microglial cultures were prepared as previously described [23]. Briefly, cortices were harvested from postnatal day 2–3 (P2–P3) Lewis rat pups, minced and incubated with trypsin/EDTA (Mediatech Manassas, VA). The supernatant was then replaced with DMEM (Mediatech, Manasses VA) supplemented with 10% fetal bovine serum (Hyclone Logan, UT), 1.1% GlutaMax (Invitrogen Carlsbad, CA), and 1% penicillin/streptomycin (100 U/ml penicillin, 100 µg/ml streptomycin, Mediatech, Manassas, VA) containing 2000 units DNase (Sigma St Louis, MO). The tissue was mechanically disrupted by trituration, the cell suspensions were centrifuged, and the cells resuspended in media without DNase. A small aliquot of cells were stained for trypan blue exclusion for counting, then cells were plated at 1×10^6^ cells per 75 cm^2^ flask. Cultures were maintained at 37°C with 5% CO_2_, and media was changed every 3–4 days. After 10 days *in vitro* (DIV 10) microglia were harvested by gently shaking the flasks by hand for 1 minute. The resulting cells were found to be >98% microglia by staining with CR3/CD11b antibody (generous gift from Dr William F. Hickey). Cells were used immediately for migration and western experiments. Peripheral macrophages were obtained from peritoneal lavage of adult rats. Briefly, 10 ml of cold PBS (Mediatech Manassas, VA) was injected into the rat peritoneum, the stomach was massaged, and fluid was subsequently removed. Cells were centrifuged and pellets were resuspended in complete media. Resuspended cells were plated in a 75 mm^2^ flask and washed the next day, leaving adherent macrophages on the flask. The resulting cells were found to be >98% macrophages by staining with CD11b antibody (mouse mAb clone WT.5; BD Franklin Lakes, NJ). CNS-1 cultured media was obtained by culturing 3×10^5^ CNS-1 cells in 500 µL of DMEM for 3 days, and then collecting the supernatant.

### Migration

The optimal experimental procedures for microglial migration in Costar Transwell plates have been previously reported [23], [24]. Cell migration was studied using Costar Transwell plates (6.5 mm diameter insert, 8.0 µm pore size, polycarbonate membrane, Corning Sparks, MD). Briefly, CNS-1 cells were plated at a density of 3×10^5^ cells per 500 µl in the bottom wells 3 days prior to the migration experiment. Microglia were harvested as described above, counted, and resuspended in serum-free media at 1×10^5^ cells per 100 µl, placed in siliconized low-adhesion microcentrifuge tubes, and treated with PPF (0.01 µM–100 µM) for 2-hour. Microglia undergoing siRNA transfection was first plated, transfected and migration was performed the following day. Cells were counted post-treatment with trypan blue to ensure survival (>99% viability) and then added (1×10^5^ cells per 100 µl) to the top chamber of a transwell plate with fibronectin-coated membranes and 500 µl of CNS-1 cells in the bottom well. After 2 hour incubation, any cells remaining on top of the membrane were washed. The membranes were rinsed with PBS, the migrated cells were fixed with 2% formaldehyde in PBS, permeabilized with 0.01% Triton X-100 (Sigma St Louis, MO) in PBS, stained with crystal violet (Sigma St Louis, MO), and rinsed twice with dH_2_O. The membranes were then dried, inverted, and mounted on microscope slides for analysis. Images of 10 random fields (20× magnification) for each membrane were captured at room temperature via a Q-fired cooled CCD camera attached to an Olympus microscope and counted by hand with aid of SigmaScan Pro imaging analysis software (SigmaScan Chicago, IL). Counts from all 10 fields were averaged to give a mean cell count for each membrane. All experiments were performed at least three times with *n* = 3 per trial. Results are expressed as mean cell migration relative to vehicle control ± S.D.

### Flow Cytometry

For flow cytometry analysis on rat tumors, rats were first perfused with cold PBS and the tumors and cortices were removed. Tissue was minced with scalpels, spun down at 1250 rpm for 10 minutes and then resuspended in digestion media (10 mL HBSS, 2,000 U DNase, 400 µL Collagenase D). Tissue was incubated in a 37°C water bath for 40 minutes and then spun down (1250 rpm for 10 minutes). Tissue was resuspended in Percoll media (10.5 mL HBSS and 4.5 mL Percoll) and spun down for 30 minutes at 2000 rpm on slow acceleration and deceleration. Fc receptors were blocked using FBS for 15 minutes before staining. Cells were first stained with anti-TROY for 1 hr on ice in PBS. Cells were washed with cold PBS and then stained with anti-CD45 PE 1∶200 (BD Pharmingen San Jose, CA), anti-CDllb-FITC 1∶100 (BD Pharmingen San Jose, CA) and anti-rabbit APC 1∶100 (Santa Cruz Santa Cruz, CA) on ice in the dark for 1 hr. Cells were washed and then analyzed. For microglia cells, cells were collected then incubated at 37°C in 24-well plates (Falcon Franklin Lakes, NJ) at a concentration of 3×10^5^ cells/well with CNS-1 supernatant for 1 hr with PPF (0.01 µM- 10 µM). Cells were then trypsinized, washed and stained on ice in PBS for 30 minutes with anti-CDllb FITC. Fc receptors were blocked using FBS for 15 minutes before staining. All flow cytometry experiments were performed on a FACSCanto (BD Bioscience Franklin Lakes, NJ).

### Western Blot Analysis

CNS-1 cells, microglia, or macrophages were plated at a cell density of 3×10^5^ cells/well in a 12-well plate (Falcon Franklin Lakes, NJ) and treated with PPF (0.01 µM–10 µM) or PBS when needed. The cells were then collected with Lamelli buffer and beta mercapto-ethanol and the protein was then quantified using the Lowry method (DC Assay, Bio-Rad Hercules, CA). Protein (40 mg) and a standard marker were subjected to SDS-PAGE (10% gels, Bio-Rad), transferred to PVDF membranes (Bio-Rad Hercules, CA), and blocked with 5% milk in TBS-Tween 20 (0.05%, Sigma St Louis, MO). The membranes were probed with rabbit anti-TROY (1∶20, AbCam Cambridgeshire, UK), rabbit anti-Pyk2 (1∶500, AbCam Cambridgeshire, UK), rabbit anti-pJNK (1∶500, BD Pharmingen San Jose, CA), rabbit anti-JNK (1∶1000, BD Pharmingen San Jose, CA) or mouse anti-Rac1 (1∶250, AbCam Cambridgeshire, UK) primary antibody for 16 hours at 4°C. Membranes were washed three times, and then incubated with goat anti-rabbit HRP-conjugated secondary (1∶300) antibody or anti-mouse HRP-conjugated secondary (1∶300) for 1 hour at 22°C. Visualization was done with SuperSignal West Femto Maximum Sensitivity Substrate (Pierce Rockford, IL) for 5 minutes and imaged using the Syngene G-Box (Synoptics Frederick, MD). Cellular suspensions were analyzed by stripping membranes and reprobing them with mouse monoclonal anti-β-actin primary antibody (1∶1000, Abcam Cambridgeshire, UK). Band intensities were quantified using the analysis software provided with the Syngene G-Box. The relative intensity of bands were divided by the intensity of the β-actin band and then compared to control. Data are expressed as fold change in band intensity normalized to naïve control ± S.D.

### Small interference RNA knockdown

Small interference RNA (siRNA) oligonucleotides specific for TROY (#1:s144862, #2:s144863, #3:s144864) were validated by and purchased from Invitrogen (Grand Island, NY). Transient transfection was carried out using iFect (Neuromics Edina, MN) as previously described [25]. Briefly, microglia were plated at 3×10^5^ cells/well in a 12-well plate. Once cells had adhered, they were transfected with 1 µg siRNA. Control samples were treated with empty vector siRNA (Sigma St Louis, MO) or iFect reagent alone. Cells were left in microglia media (10% fetal bovine serum (Hyclone Logan, UT), 1.1% GlutaMax (Invitrogen Carlsbad, CA), and 1% penicillin/streptomycin (100 U/ml penicillin, 100 µg/ml streptomycin, Mediatech, Manassas, VA)) at 37°C with 5% CO_2_ overnight and then used the following day for experiments.

## Results

### TROY is expressed in microglia in response to CNS-1 tumor stimulation

**Figure 1:**
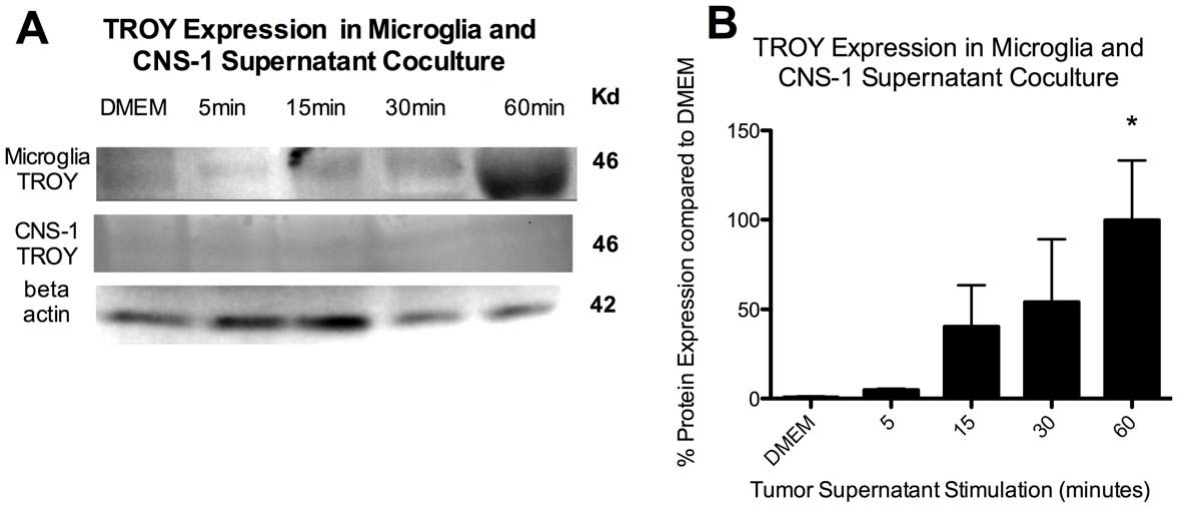
TROY is expressed in microglia in response to CNS-1 tumor stimulation. (A) TROY western blot of microglia cultured with CNS-1 conditioned media over time. (B) A graphical representation of differential TROY expression in microglia cultured with CNS-1 conditioned media over time (* = *p*<0.05). Graph is representative of three western blots from three replicates.

We sought to investigate if TROY may be involved in microglial recruitment to the tumor site. We first determined whether microglia express TROY. Microglia display low levels of TROY expression when cultured in DMEM, however, TROY expression is significantly (p<0.05) increased in response to CNS-1 conditioned media (Fig. 1). After 1 hour, TROY expression was increased by over 100%. Meanwhile, TROY expression was not detected by western blot in peritoneal macrophages in response to CNS-1 conditioned media (data not shown) nor in CNS-1 tumor cells (Fig. 1).

### Microglia increase TROY signaling molecules in response to CNS-1 conditioned media

To further investigate TROY signaling we asked whether Pyk2, Rac1 and pJNK are increased in microglia in response to CNS-1 conditioned media after the observed increase in TROY expression. Pyk2 and Rac1 have recently been reported to be involved in TROY expression in human glioma cell lines and both proteins are involved in cellular migration and invasion. The protein kinase, pJNK, is further downstream and has also been linked to TROY expression.

**Figure 2:**
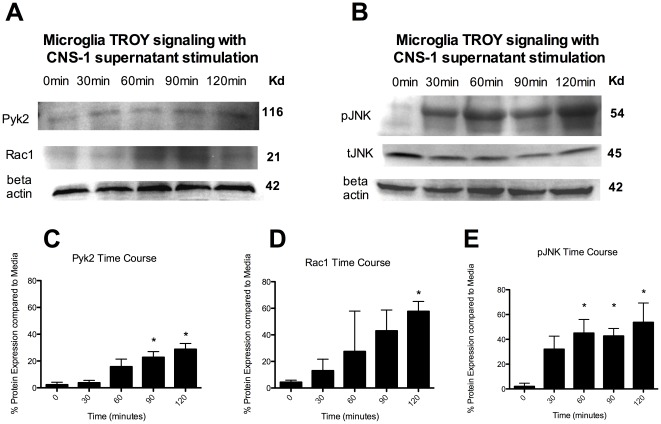
Microglia increase Pyk2, Rac1 and pJNK expression in response to CNS-1 conditioned media. (A) Pyk2 and Rac1 western blot of microglia cultured with CNS-1 conditioned media over time. (B) pJNK and tJNK western blot of microglia cultured with CNS-1 conditioned media over time. (C) A graphical representation of differential pyk2 expression in microglia cultured with CNS-1 conditioned media over time (* = *p*<0.05). (D) A graphical representation of differential Rac1 expression in microglia cultured with CNS-1 conditioned media over time (* = *p*<0.05). (E) A graphical representation of differential pJNK expression in microglia cultured with CNS-1 conditioned media over time (* = *p*<0.05). Graphs are representative of three western blots from three replicates.

Microglia were cultured with CNS-1 conditioned media over time and changes in protein expression were analyzed by western blot analyses. Both Pyk2 and Rac1 significantly increased expression over time (p<0.05) (Fig. 2A,C,D). Pyk2 was significantly increased by 90 minutes compared to time 0, while Rac1 was significantly increased at 120 minutes (Fig. 2D). The tyrosine kinase, pJNK was significantly increased at 30 minutes compared to time 0, with a further increase at 60 minutes (P<0.05) (Fig. 2B,E). There was no significant change in tJNK (Fig. 2E).

### PPF decreases both TROY expression and downstream signaling of Pyk2, Rac1 and pJNK in microglia

**Figure 3:**
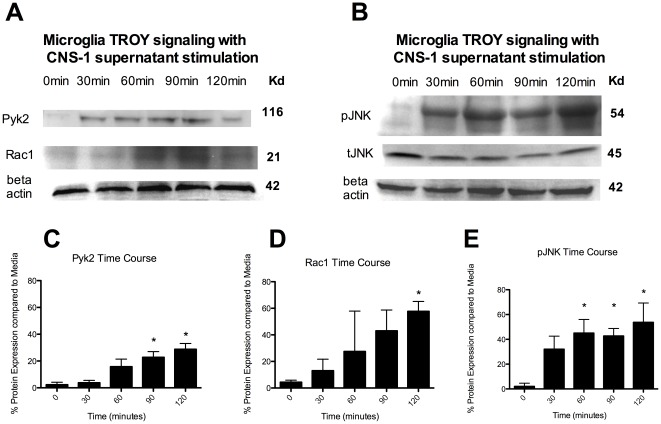
Propentofylline decreases TROY expression in microglia. (A) TROY western blot of microglia cultured with CNS-1 conditioned media and treated with PPF. (B) A graphical representation of differential TROY expression microglia cultured with CNS-1 conditioned media and treated with PPF (* = *p*<0.05). Graph is representative of three western blots from three replicates.

As mentioned previously, PPF has a direct mechanism on microglial migration. It has also been previously reported that PPF decreases integrin CD11b expression (Figure S1) [19]. Recognizing TROY's shared downstream signaling with integrins (Pyk2, Rac1) and PPF's inhibition of microglial migration, we asked if TROY is a target for PPF. Microglia were co-cultured *in vitro* with CNS-1 conditioned media for 1 hour and treated with PPF (0.01 µM–10 µM). PPF significantly decreased TROY expression in microglia in a dose dependent manner (p<0.05) (Fig. 3), with decreased expression by almost 50% at 10 µM (Fig. 3).

**Figure 4:**
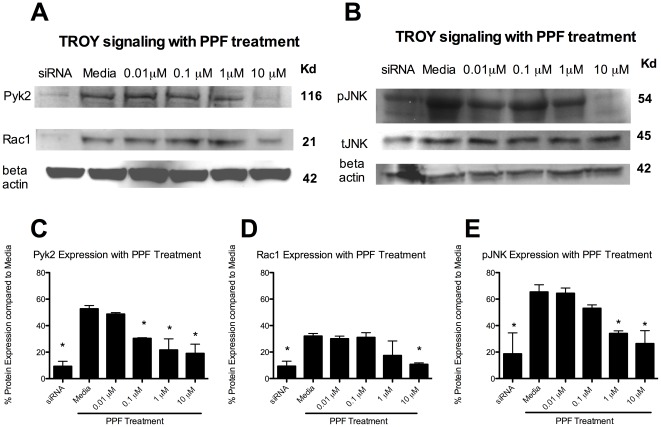
Propentofylline decreases Pyk2, Rac1 and pJNK. (A) Pyk2, Rac1 and pJNK western blot of microglia cultured with CNS-1 conditioned media and treated with PPF. (B) pJNK and tJNK western blot of microglia cultured with CNS-1 conditioned media and treated with PPF. (C) A graphical representation of differential pyk2 expression in microglia cultured with CNS-1 conditioned media and treated with PPF (* = *p*<0.05). (D) A graphical representation of differential Rac1 expression in microglia cultured with CNS-1 conditioned media and treated with PPF (* = *p*<0.05). (E) A graphical representation of differential pJNK expression in microglia cultured with CNS-1 conditioned media and treated with PPF (* = *p*<0.05). Graphs are representative of three western blots from three replicates.

Next, Pyk2, Rac1 and pJNK expression with PPF treatment was assessed. Microglia were cultured with CNS-1 conditioned media and treated with PPF for 90 minutes. Microglia treated with TROY siRNA was used as a positive control for inhibition of the TROY signaling pathway in microglia. TROY siRNA treatment significantly (p<0.05) decreased Pyk2, Rac1 and pJNK signaling, as expected. PPF significantly inhibited Pyk2 with a dose as low as 0.1 µM compared to media (p<0.05) (Fig. 4A,C). Rac1 was significantly inhibited with PPF treatment; however, this was at a dose of 10 µM (Fig. 4A,D). PPF further inhibited pJNK expression in microglia compared to media at both 1 µM and 10 µM (Fig. 4E). There was no decrease in tJNK with PPF or siRNA blockade.

### TROY knockdown results in a decrease of microglia migration towards CNS-1 tumor cells

**Figure 5:**
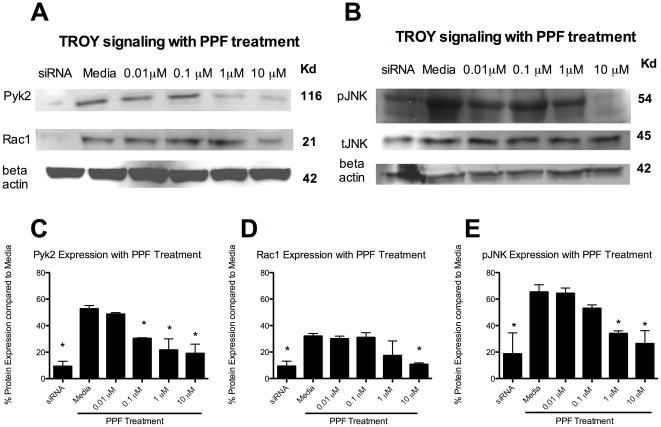
TROY knockdown results in a decrease of microglial migration towards CNS-1 tumor cells. (A) Western blot demonstrating decreased TROY expression in microglia cultured with CNS-1 conditioned media and treated with TROY siRNA. (B) Microglia were treated with TROY siRNA, and then migrated towards CNS-1 cells. Migration of microglia in response to CNS-1 cells is significantly decreased compared to media (* = *p*<0.05).

To determine the functional role of TROY in microglia, we used siRNA to knock down TROY expression in primary microglia (Fig. 5A). Three different siRNAs were tested. The siRNA #2 demonstrated the strongest knockdown and was used for Boyden chamber migration experiments. Negative siRNA and iFect reagent were used as controls while 10 µM PPF was used as a positive control for inhibition of microglial migration. Migration of microglia towards CNS-1 cells was significantly inhibited (80% decrease in migration) compared to media and negative siRNA (p<0.05). Inhibition of migration with TROY knock down was comparable to 10 µM PPF. There was no statistical difference between TROY knockdown and PPF.

### TROY is expressed in tumor infiltrating microglia in vivo and decreased by propentofylline

**Figure 6:**
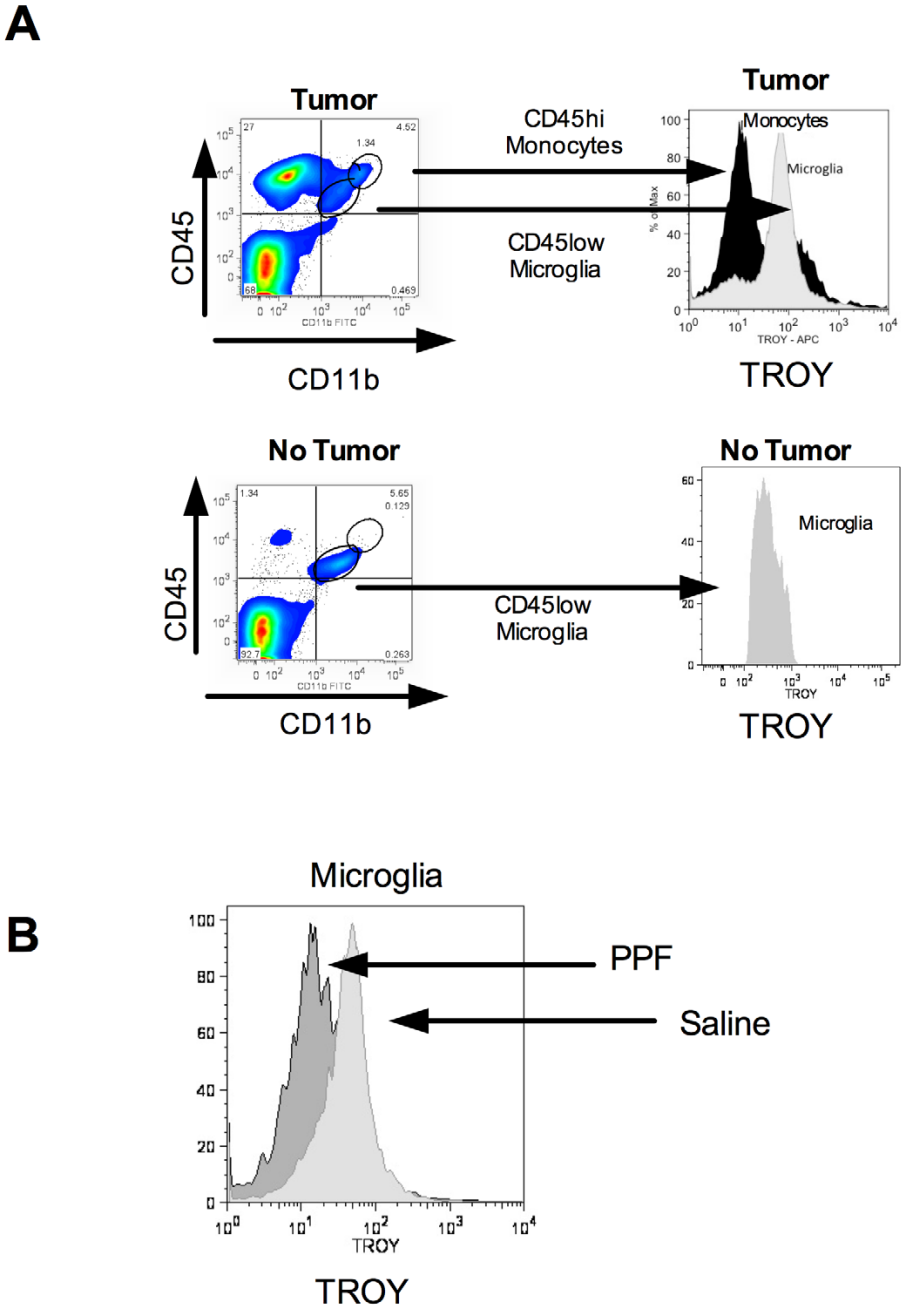
TROY is expressed in tumor infiltrating microglia, not macrophages. (A) Lewis rats were implanted with 3×10^5^ CNS-1 cells. Tumors were collected on day 10 from tumor bearing rats and untreated rats, and then stained for microglia (CD45^+lo^, CDllb^+^), macrophages (CD45^+hi^, CDllb^+^) and TROY. Gating is shown in figure, histogram is representative of 6 rats/group. (B) Lewis rats were implanted with CNS-1 cells and then treated with 50 mg/kg PPF or saline on day 8 for 2 days. Tumors were collected and stained for microglia (CD45^+lo^, CDllb^+^), macrophages (CD45^+hi^, CDllb^+^) and TROY. Histogram is representative of 3 rats/group.

To confirm TROY expression *in vivo*, Lewis rats were inoculated with 3×10^5^ CNS-1 cells. Lewis rats without tumors were used as a control. At day 10, rats were euthanized and tumors or cerebellum from non-tumor rats were stained for microglia (CD45^+lo^, CDllb^+^), monocytes (CD45^+hi^, CDllb^+^) and TROY (Fig. 6A). TROY was highly expressed in tumor infiltrating microglia compared to monocytes (Fig. 6A). Furthermore, microglia isolated from non-tumor rats displayed minimal TROY expression (Fig. 6A).

To study PPF's effects on TROY in vivo, rats were inoculated with CNS-1 cells and tumors were grown for 8 days. Rats were then treated with 50 mg/kg of PPF or saline i.p. daily for two days. On day 10, rats were euthanized and tumors were stained for microglia (CD45^+lo^, CDllb^+^), monocytes (CD45^+hi^, CDllb^+^) and TROY. PPF decreased expression of TROY in tumor infiltrating microglia compared to saline treated rats (Fig. 6B).

## Discussion

We demonstrate in these studies that 1) TROY is expressed in microglia in response to CNS-1 cells, 2) Pyk2, Rac1 and pJNK are downstream signaling molecules of TROY in microglia 3) PPF decreases TROY expression and its downstream molecules and 4) TROY is involved in microglia migration towards CNS-1 cells.

Glioma has a high infiltrative population of microglia and peripheral macrophages to the tumor site [3]. The infiltrative property of gliomas is cited as a key reason for low survival and tumor recurrence [26]. It has been established that these infiltrative immune cells have a direct role in increasing glioma cell invasion and growth [8], [27]. The recent past has been witness to promising targeted therapies for glioma, unfortunately these single targets have demonstrated unpromising results in clinical trials in GBM patients [28]. The failure to also target the strong tumor promoting microenvironment is a possible reason for the lack of success. Our previous work identified PPF as a novel drug for GBM that targets microglia in the tumor microenvironment [9]. This paper sought to further identify the mechanism of PPF's actions.

In this study we demonstrate that PPF decreases the TNFRSF member TROY, which is involved in microglial migration towards CNS-1 cells. TROY is a unique TNFRSF member whose expression is strictly controlled [29]. TROY is highly expressed in embryonic development, and then minimally expressed in the adult, indicating that continual expression is unfavorable [30], [31]. However, TROY is expressed once again in microglia in response to CNS-1 cells, suggesting this receptor plays a crucial role in microglial migration towards tumor cells, ultimately effecting glioma growth and invasion. Our studies in microglia were conducted in a CNS-1 rodent glioma model. This rodent model does not express TROY in the CNS-1 tumor cells, which enabled specific studies of TROY expression in the tumor microenvironment. However, animal models do not fully recapitulate the genetic diversity of the human disease and TROY expression in microglia and infiltrating macrophages needs to be confirmed in human glioma tissue. Although TROY is not expressed in CNS-1 cells, expression has been cited in human glioma cell lines [15]. If TROY expression is confirmed in human microglia, this may represent a novel biomarker, which is expressed in both glioma tumor cells and the tumor microenvironment.

We identify Pyk2, Rac1 and pJNK as downstream signaling molecules for TROY. Pyk2 and Rac1 are increased after 90 minutes and 120 minutes in microglia cultured in CNS-1 conditioned media. This is after the 60 minute time point when TROY expression is increased. The tyrosine kinase pJNK has an initial increase at 30 minutes before TROY is increased. However, there is a second increase in expression at 60 minutes, which correlates with TROY expression. Other signaling molecules known to increase pJNK, such as PDGF, can explain the initial increase in pJNK expression at time 30 minutes [26]. The TROY pathway was further confirmed by siRNA knockdown of TROY in microglia, which demonstrated a decrease in Pyk2, Rac1 and pJNK. All three of these molecules have been linked to migration and invasion, further supporting TROY's role in microglial migration. It is still unknown what in the CNS-1 conditioned media initiates TROY expression or what the ligand is that signals through TROY in microglia. Future experiments will focus on identifying signaling molecules from CNS-1 cells that promote TROY expression and signaling in microglia.

Most research studying microglia and infiltrative macrophages in the glioma microenvironment do not differentiate these two populations. To date there is not a single marker that can easily distinguish the two monocyte populations. Currently, staining based on CD45 expression (high vs low) is the best method, however, the gating of these two populations can often be subjective. In this paper we demonstrate TROY as a novel marker that differentiates microglia and macrophages in the tumor microenvironment. It remains to be investigated if this molecule differentiates microglia and macrophages in human tissue or in other disease states. However, it does provide evidence of differential function and supports further investigation into how each of these two populations may differentially affect glioma tumor growth.

In summary, we present TROY as a novel receptor expressed in microglia in response to CNS-1 cells, involved in microglia migration, and targeted by propentofylline.

## Supporting Information

Figure S1 **Histogram representation of CDllb expression in microglia with PPF treatment.** Microglia were cultured in vitro with CNS-1 conditioned media for 60 minutes then treated with propentofylline for another 60 minutes. CDllb expression was analyzed by FACS. Propentofylline decreased expression of CDllb with increasing dosages. Histogram is representative image of n = 3.
(TIF)

## References

[1] DeAngelis LM (2005). Chemotherapy for brain tumors–a new beginning.. N Engl J Med.

[2] Ohgaki H, Kleihues P (2005). Population-based studies on incidence, survival rates, and genetic alterations in astrocytic and oligodendroglial gliomas.. J Neuropathol Exp Neurol.

[3] Roggendorf W, Strupp S, Paulus W (1996). Distribution and characterization of microglia/macrophages in human brain tumors.. Acta Neuropathol.

[4] Yang I, Han SJ, Kaur G, Crane C, Parsa AT (2010). The role of microglia in central nervous system immunity and glioma immunology.. J Clin Neurosci.

[5] Tuettenberg J, Grobholz R, Seiz M, Brockmann MA, Lohr F (2009). Recurrence pattern in glioblastoma multiforme patients treated with anti-angiogenic chemotherapy.. J Cancer Res Clin Oncol.

[6] Gabrusiewicz KE-MA, Lipko M, Sielska M, Frankowska M, Kaminska B (2011). Characteristics of the alternative phenotype of microglia/macrophages and its modulation in experimental gliomas.. PLoS One.

[7] Wu A, Wei J, Kong LY, Wang Y, Priebe W (2010). Glioma cancer stem cells induce immunosuppressive macrophages/microglia.. Neuro Oncol.

[8] Sliwa MMD, Gabrusiewicz K, Synowitz M, Glass R, Zawadzka M, Wesolowska A, Kettenmann H, Kaminska B (2007). The invasion promoting effect of microglia on glioblastoma cells is inhibited by cyclosporin A.. Brain.

[9] Jacobs VL, Landry RP, Liu Y, Romero-Sandoval EA, De Leo JA (2011). Propentofylline decreases tumor growth in a rodent model of glioblastoma multiforme by a direct mechanism on microglia.. Neuro Oncol.

[10] D'Abaco GM, Kaye AH (2007). Integrins: molecular determinants of glioma invasion.. J Clin Neurosci.

[11] Aplin AE, Howe A, Alahari SK, Juliano RL (1998). Signal transduction and signal modulation by cell adhesion receptors: the role of integrins, cadherins, immunoglobulin-cell adhesion molecules, and selectins.. Pharmacol Rev.

[12] Mariani L, Beaudry C, McDonough WS, Hoelzinger DB, Demuth T (2001). Glioma cell motility is associated with reduced transcription of proapoptotic and proliferation genes: a cDNA microarray analysis.. J Neurooncol.

[13] Hoelzinger DB, Mariani L, Weis J, Woyke T, Berens TJ (2005). Gene expression profile of glioblastoma multiforme invasive phenotype points to new therapeutic targets.. Neoplasia.

[14] Veroni C, Gabriele L, Canini I, Castiello L, Coccia E (2010). Activation of TNF receptor 2 in microglia promotes induction of anti-inflammatory pathways.. Mol Cell Neurosci.

[15] Paulino VM, Yang Z, Kloss J, Ennis MJ, Armstrong BA (2010). TROY (TNFRSF19) is overexpressed in advanced glial tumors and promotes glioblastoma cell invasion via Pyk2-Rac1 signaling.. Mol Cancer Res.

[16] Park JB, Yiu G, Kaneko S, Wang J, Chang J (2005). A TNF receptor family member, TROY, is a coreceptor with Nogo receptor in mediating the inhibitory activity of myelin inhibitors.. Neuron.

[17] Shao Z, Browning JL, Lee X, Scott ML, Shulga-Morskaya S (2005). TAJ/TROY, an orphan TNF receptor family member, binds Nogo-66 receptor 1 and regulates axonal regeneration.. Neuron.

[18] Qiu W, Hu Y, Andersen TE, Jafari A, Li N (2010). Tumor necrosis factor receptor superfamily member 19 (TNFRSF19) regulates differentiation fate of human mesenchymal (stromal) stem cells through canonical Wnt signaling and C/EBP.. J Biol Chem.

[19] Sweitzer S, De Leo J (2011). Propentofylline: glial modulation, neuroprotection, and alleviation of chronic pain.. Handb Exp Pharmacol.

[20] Nagata K, Ogawa T, Omosu M, Fujimoto K, Hayashi S (1985). In vitro and in vivo inhibitory effects of propentofylline on cyclic AMP phosphodiesterase activity.. Arzneimittelforschung.

[21] Fredholm BB, Lindstrom K (1986). The xanthine derivative 1-(5′-oxohexyl)-3-methyl-7-propyl xanthine (HWA 285) enhances the actions of adenosine.. Acta Pharmacol Toxicol (Copenh).

[22] Kruse CA, Molleston MC, Parks EP, Schiltz PM, Kleinschmidt-DeMasters BK (1994). A rat glioma model, CNS-1, with invasive characteristics similar to those of human gliomas: a comparison to 9 L gliosarcoma.. J Neurooncol.

[23] Nutile-McMenemy N, Elfenbein A, Deleo JA (2007). Minocycline decreases in vitro microglial motility, beta1-integrin, and Kv1.3 channel expression.. J Neurochem.

[24] Horvath RJ, Nutile-McMenemy N, Alkaitis MS, Deleo JA (2008). Differential migration, LPS-induced cytokine, chemokine, and NO expression in immortalized BV-2 and HAPI cell lines and primary microglial cultures.. J Neurochem.

[25] Tsui CC, Shankland SJ, Pierchala BA (2006). Glial cell line-derived neurotrophic factor and its receptor ret is a novel ligand-receptor complex critical for survival response during podocyte injury.. J Am Soc Nephrol.

[26] Pollard JW (2004). Tumour-educated macrophages promote tumour progression and metastasis.. Nat Rev Cancer.

[27] Yeh WL, Lu DY, Liou HC, Fu WM (2012). A forward loop between glioma and microglia: Glioma-derived extracellular matrix-activated microglia secrete IL-18 to enhance the migration of glioma cells.. J Cell Physiol.

[28] Gilbert MR (2011). Recurrent glioblastoma: a fresh look at current therapies and emerging novel approaches.. Semin Oncol.

[29] Eby MT, Jasmin A, Kumar A, Sharma K, Chaudhary PM (2000). TAJ, a novel member of the tumor necrosis factor receptor family, activates the c-Jun N-terminal kinase pathway and mediates caspase-independent cell death.. J Biol Chem.

[30] Hu S, Tamada K, Ni J, Vincenz C, Chen L (1999). Characterization of TNFRSF19, a novel member of the tumor necrosis factor receptor superfamily.. Genomics.

[31] Morikawa Y, Hisaoka T, Kitamura T, Senba E (2008). TROY, a novel member of the tumor necrosis factor receptor superfamily in the central nervous system.. Ann N Y Acad Sci.

