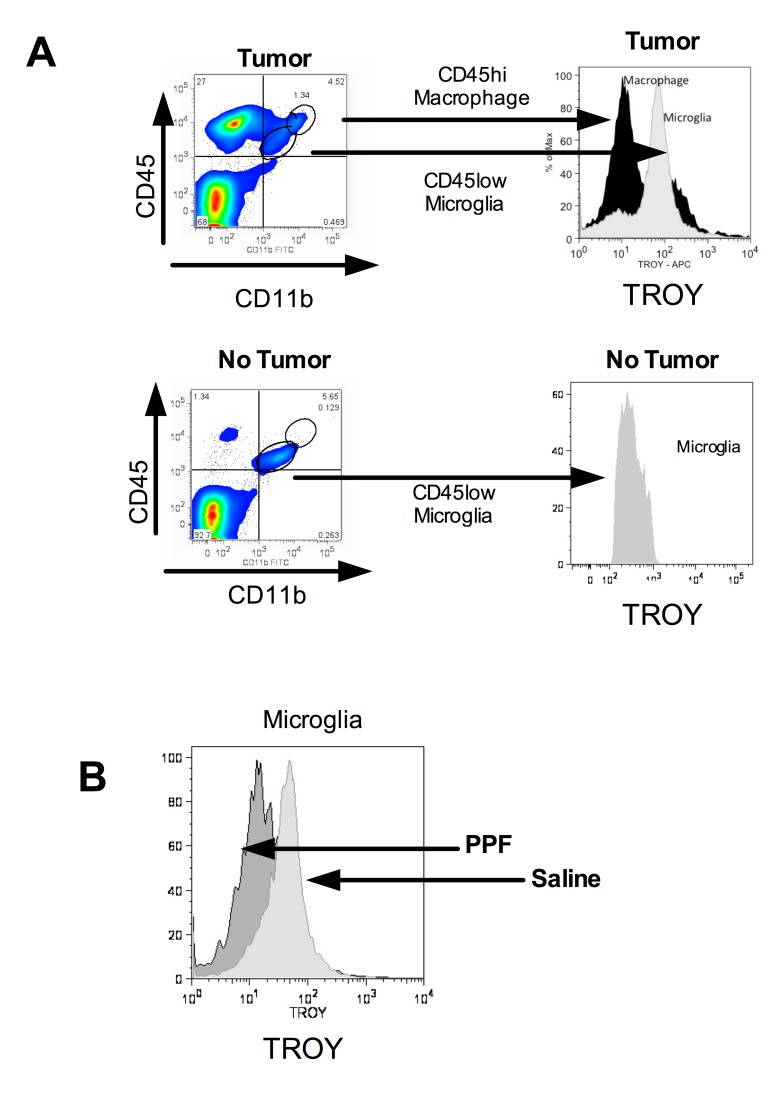# Correction: Propentofylline Targets TROY, a Novel Microglial Signaling Pathway

**DOI:** 10.1371/annotation/d8f0ef0f-414e-49aa-92a8-fd8d838b611b

**Published:** 2013-01-17

**Authors:** Valerie L. Jacobs, Yingna Liu, Joyce A. De Leo

There were errors in the published figures of this article. Please see the correct figures here:

Figure 1: 

**Figure:**
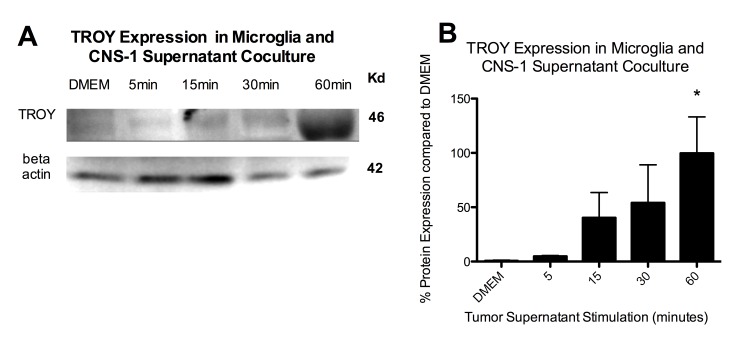


Figure 2: 

**Figure:**
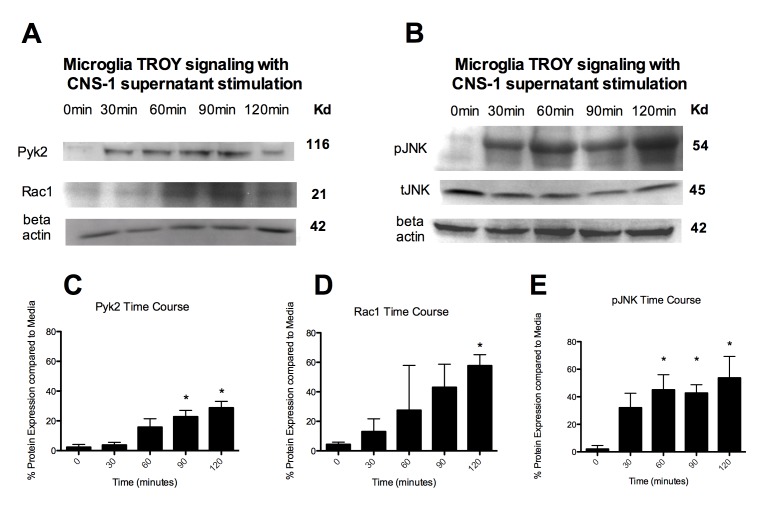


Figure 3: 

**Figure:**
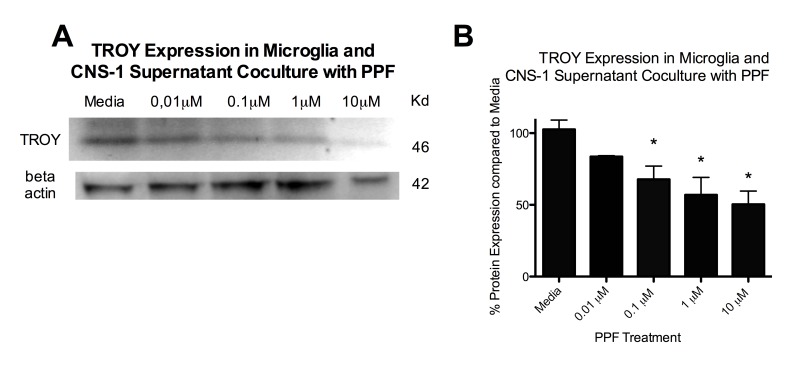


Figure 4: 

**Figure:**
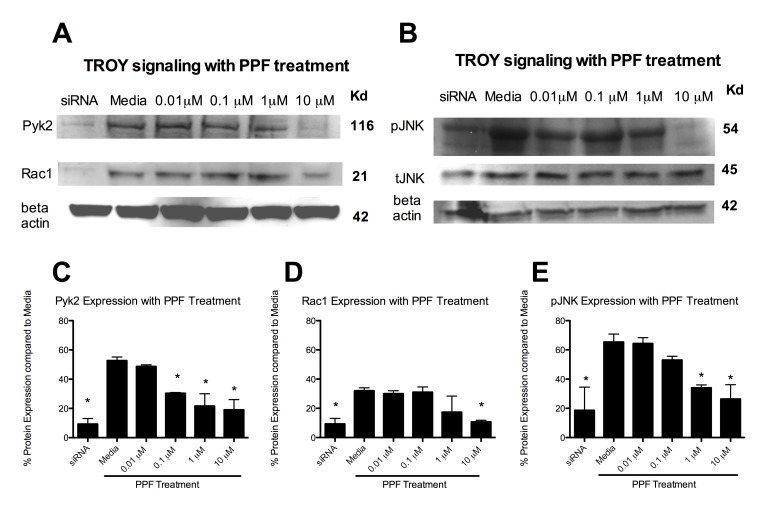


Figure 5: 

**Figure:**
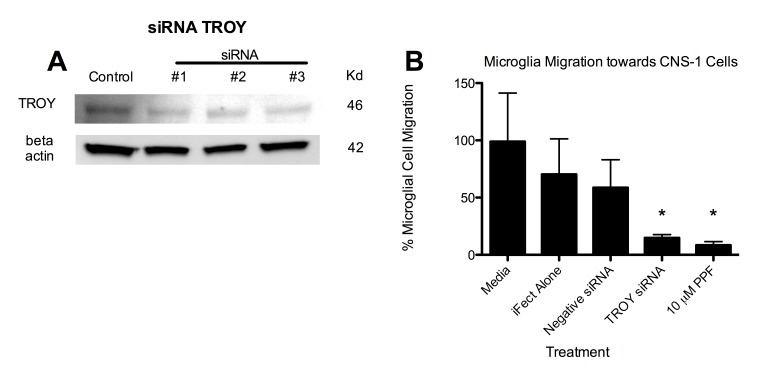


Figure 6: 

**Figure:**