# Supplementary figures and images for: Propentofylline Targets TROY, a Novel Microglial Signaling Pathway
Source: PLoS One. 2012 May 23;7(5):e37955. doi: 10.1371/journal.pone.0037955 (PMC3359343; doi:10.1371/journal.pone.0037955)

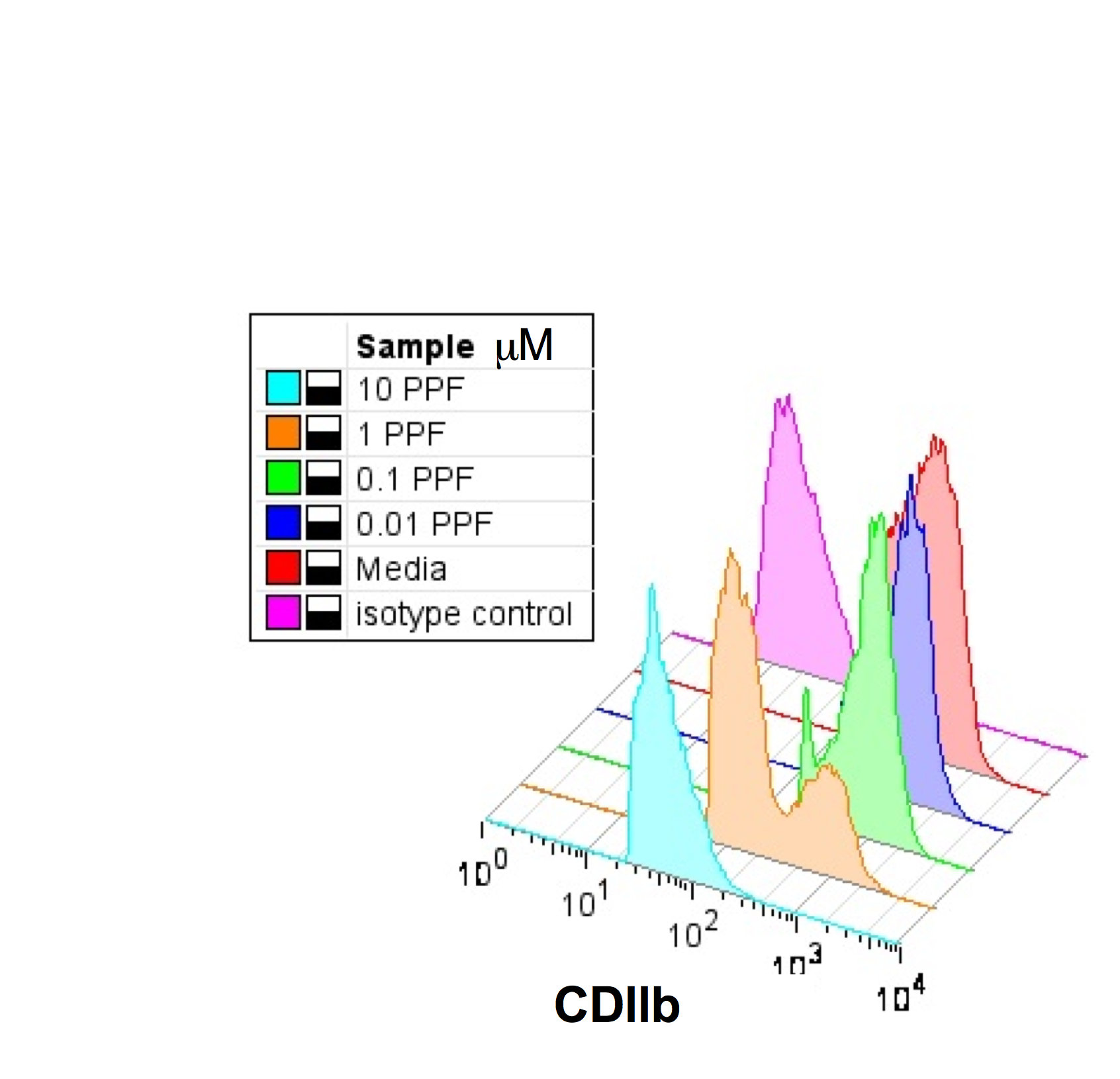

Supplement: Figure S1 — Histogram representation of CDllb expression in microglia with PPF treatment. Microglia were cultured in vitro with CNS-1 conditioned media for 60 minutes then treated with propentofylline for another 60 minutes. CDllb expression was analyzed by FACS. Propentofylline decreased expression of CDllb with increasing dosages. Histogram is representative image of n = 3. (TIF) [file pone.0037955.s001.tif]
